# Cancer Stem Cells and Radioresistance: Rho/ROCK Pathway Plea Attention

**DOI:** 10.1155/2016/5785786

**Published:** 2016-08-15

**Authors:** Annapurna Pranatharthi, Cecil Ross, Sweta Srivastava

**Affiliations:** ^1^St. John's Medical College Hospital, Bangalore 560034, India; ^2^National Centre for Biological Sciences, Bangalore 560065, India

## Abstract

Radiation is the most potent mode of cancer therapy; however, resistance to radiation therapy results in tumor relapse and subsequent fatality. The cancer stem cell (CSC), which has better DNA repair capability, has been shown to contribute to tumor resistance and is an important target for treatment. Signaling molecules such as Notch, Wnt, and DNA repair pathways regulate molecular mechanisms in CSCs; however, none of them have been translated into therapeutic targets. The RhoGTPases and their effector ROCK-signaling pathway, though important for tumor progression, have not been well studied in the context of radioresistance. There are reports that implicate RhoA in radioresistance. ROCK2 has also been shown to interact with BRCA2 in the regulation of cell division. Incidentally, statins (drug for cardiovascular ailment) are functional inhibitors of RhoGTPases. Studies suggest that patients on statins have a better prognosis in cancers. Data from our lab suggest that ROCK signaling regulates radioresistance in cervical cancer cells. Collectively, these findings suggest that Rho/ROCK signaling may be important for radiation resistance. In this review, we enumerate the role of Rho/ROCK signaling in stemness and radioresistance and highlight the need to explore these molecules for a better understanding of radioresistance and development of therapeutics.

## 1. Introduction

Radioresistance and relapse are a burden for cancer therapy. Despite best efforts and technological advances, there is a considerable percentage of patients who do not respond to therapy. Most importantly, all the therapies currently in practice have severe side effects. As there are no means of treating relapse or metastatic tumors, patients are usually advised on palliative care. The diagnosis and treatment in the advanced stages are costly exercises with poor prognosis and compliance. Similarly, recurrent tumors also demonstrate poor prognosis. It is important to note that these therapies have severe side effects on the patients; thus, it is essential to stratify the patients who are most likely to respond to therapy. The development of cancer therapy is extremely dependent on an understanding of tumor biology and predictive markers. The predictive markers define the population of patients who will respond to therapy. A solid tumor is a highly complex system comprising of proliferating tumor cells, blood vessels, lymphatic vessels, and nontumor cells. The cross talk between these cell types and regulation by microenvironment is essential for tumor progression and also increases the complexity of treatment. Tumor heterogeneity, by virtue of the presence of different cell subpopulations, plays a major role in differential therapy response. One such subset which has garnered the substantial attention of the scientific community is the cancer-initiating cells (CICs) or cancer stem cell (CSC). One of the first observations on CSCs was published by Dr. Richards in 1955. He demonstrated the existence of a stem cell pool using deoxyribose nucleic acid measurements in Ehrlich's and Krebs's ascites tumor. He observed that only a small fraction of cells was capable of normal and regular mitosis which could be due to the division of the stem cells [1]. The cancer stem cell model argues that it is the major contributing factor to disease progression and therapy response as they have the capability to self-renew and generate heterogeneous lineages of cancer cells [2, 3].

One of the most convincing evidences for the existence of CSCs first surfaced from studies by Bonnet and Dick, who suggested that there is a relatively small population (0.01–1%) of cells that have enhanced tumorigenic properties [4]. CD34+/CD38− cells purified from patients with acute myeloid leukaemia were able to generate tumors in NOD/SCID mice that were histologically similar to the donor. The existence of CSCs has also been reported in several tumors including brain [5], breast [6], colon [7], ovary [8], pancreas [9], and prostate [10]. Recently, the presence of CIC marked by expression of Kr19+/Lgr5− has been reported in colon cancer [11].

## 2. Radioresistance and Cancer Stem Cells

There is increasing evidence that CSCs determine the fate of tumor and its clinical outcome. It is likely that the abolition of this subset of cells may impact the clinical outcomes. Radiation therapy plays a pivotal role in the treatment of several tumors such as head and neck cancer and cervical carcinoma. However, the emergence of resistance to therapy is a major concern in the treatment of carcinomas. Such resistance may be attributed to various mechanisms that exist or remain to be identified in the stem cells.

Glioblastoma multiforme (GBM) is one of the most aggressive tumors with poor prognosis and ionizing radiation alone or adjuvant therapy is only palliative and is noncurative. In glioma, CSCs marked by CD133 expression exhibit properties of resistance to radiation [12, 13]. Similarly, Phillips et al. observed that in breast cancers CICs are relatively more radioresistant [14]. Bao et al. observed that in both cell culture and mice brain the CD133 expressing glioma cells survived radiation by activating the DNA damage checkpoint signaling. In a similar study, Gao et al. observed that fractionated radiation of a human glioblastoma cell line, U87-MG, enhanced cell division signaling pathways, which might be leading to repopulation of the cancer stem cell pool. Interestingly, both the CD133 positive and negative cells have been shown to possess the capability of inducing tumors in mice; however, there are reports which show that radiation induces enrichment of the CD133 expressing cells [15, 16].

CSCs also impact therapy outcome in non-small cell lung cancer (NSCLC). Radiotherapy is also used as a palliative care modality for NSCLC. Using NSCLC derived cell lines, A549 and H460, Gomez-Casal and groups show that cells surviving radiation treatment had enhanced stemness and epithelial-mesenchymal transition (EMT) properties. These cells overexpressed CD24, CD44, Sox2, and Oct4 along with EMT markers such as snail1 and N-cadherin [17]. The interaction of CD44 with Tiam1, a guanine exchange factor and regulator of GTPases, is important for Rac1 signaling activation, which supports cell migration, in metastatic breast cancer cells [18]. The presence of CICs with CD49f and CD133 overexpression has also been reported in cervical carcinoma cell lines. Gene profiling analysis of the cells showed transcriptional upregulation of DNA repair machinery proteins. Similarly, dose-dependent irradiation of the cervical carcinoma cell lines resulted in enrichment of the CICs [19]. These and several other such observations vehemently support that CSCs have a major role in radioresistance.

## 3. CSCs and Molecular Mechanisms Regulating Radiation Response

The role of both intrinsic and extrinsic factors in the induction of resistance has been widely debated. There are various studies which support the role of the microenvironment in regulation of CSC and its function. Similarly, there is strong evidence indicating the role of signaling molecules in the maintenance of CSCs and induction of radioresistance.

Landmark studies, in the 1990s, demonstrated that Hif-1-alpha, an oxygen sensing signaling pathway, is operational in tumor cells [20–22], and it can regulate the expression of several genes essential for tumor growth and progression [23]. Studies have also supported the role of reactive oxygen species (ROS) in radioresistance in CSCs. Diehn et al. show that CSCs contain lower ROS levels than the bulk of the tumor cells. Since ROS levels are important for radiation-induced cell death, CSCs harbouring lower levels of ROS have lesser DNA damage and thus elicit better survival [24].

**Figure 1:**
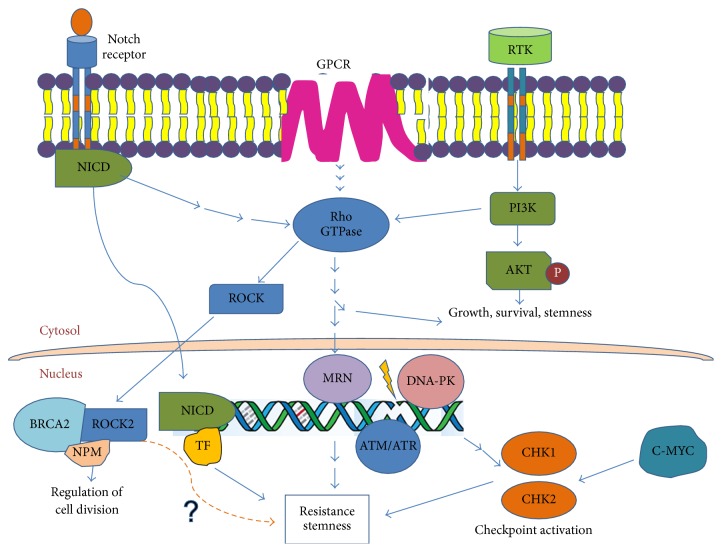
Cross talk between multiple signaling pathways involved in tumor progression and resistance.

DNA damage and repair mechanisms are widely implicated in radiation-induced effects. DNA damage is one of the earliest events after irradiation. It has been demonstrated that radioresistance in glioma cells is induced by activation of DNA repair pathway. Bao et al. show that radiation of glioma cells results in enrichment of CD133+ CSCs. Upon irradiation, these cells have activated DNA damage checkpoints and better survival rates. They demonstrated that inhibition of Chk1 and Chk2 resulted in enhanced cell death in these cells [12]. L1CAM has also been shown to induce radioresistance, by regulating NBS1, via c-Myc [25]. NBS1 is an important component of the MRN complex, involved in DNA repair. C-Myc regulates Chk1 and Chk2 by directly binding to the promoters of the two genes in nasopharyngeal carcinoma (NPC), resultantly regulating radioresistance [26]. These observations, and several others, strongly support the role of altered DNA repair mechanisms in radiation-induced resistance. There are several other signaling pathways which have important contribution to CSC maintenance (schematic representation in Figure 1). The role of Notch [27, 28], TGF-beta [29, 30], and Wnt [31, 32] in CSC maintenance has been extensively reported. These molecules are also in clinical trials to aid in radiation therapy. Hedgehog pathway inhibitors, in combination with PI3K-m-TOR inhibitors like vismodegib, sirolimus, sonidegib, and buparlisib, have worked well, both in vitro and in vivo, and are in clinical trials [33]. Clinical trials are also underway targeting Notch and Wnt pathways together. However, despite several studies, there is still a dearth of understanding of CSC and its unique molecular signatures that can be exploited for therapeutics. The GTPases have also been added to the existing plethora of molecules, contributing to radioresistance [34, 35].

## 4. RhoGTPase Signaling in Radioresistance

RhoGTPase pathway has been studied extensively in the context of tumor progression, and its effectors are reported in multiple cancers [36]. RhoGTPases are a family of conserved proteins that have been reported to be involved in cytoskeletal organisation, migration, cell division, cell adhesion, and transcriptional regulation, in which they act as a switch controlling the outcomes of these key processes, in a cell. Rho GTPases cycle between an active GTP and inactive GDP bound form, in response to signaling cues. Among them, the RhoA, RhoB, RhoC, Cdc42, and Rac1 are well-studied members of this family. The Rho GTPases require guanine exchange factors (GEFs), GTPase-activating proteins (GAPs), and Guanine Nucleotide Dissociation Inhibitors (GDIs) for the regulation of their activity, which is dependent upon GTP [36].

The Rho GTPases, in their active conformation (GTP-bound), are capable of binding effectors, for the subsequent downstream signaling activation [37, 38]. The GEFs are the proteins that facilitate the exchange of GDP to GTP on the Rho proteins, rendering them functionally active. The Net1-RhoA, ITSN-L-Cdc42, and Tiam1-Rac1 are few examples of GEFs and their respective GTPases [39–41]. GDIs bind to Rho GTPases like Ras, RhoA, and Cdc42 and play a role in the cytosolic maintenance of these Rho proteins, by sequestering the hydrophobic lipids on these molecules, thereby blocking their docking on the membrane. They also affect the binding of the GTPases to GEFs and the effector kinases [42, 43]. These proteins have an important role in tumor progression [44, 45].

RhoA has been shown to regulate tumor progression in several tumors [46–50]. Interestingly, RhoA also regulates radioresistance in glioblastoma, by modulating Survivin activity [51]. RhoA is shown to be expressed at the tumor front and found to be associated with poor prognosis in prostate cancer [48]. Inhibition of RhoA led to decreased proliferation and migration in gastric cancer cell line [49] and reduced migration in colorectal cancers [50]. Observations suggest that activated RhoA is found in the nucleus upon irradiation of tumor cells [52]. The other member, RhoB, has been reported as a tumor suppressor [53]. It has also been shown to mediate resistance in HeLa cells [54]. RhoC has been shown to regulate tumor progression, in several tumors [52, 55–60]. Interestingly, RhoC regulates several tumor phenotypes including angiogenesis, anoikis resistance, migration, invasion, and metastasis [57, 59–61]. It has been reported to induce EMT in ovarian cancer cells in response to VEGF and TGFbeta1 signaling [62]. The EMT mediated by TGFbeta1 signaling is dependent on RhoC overexpression in cervical cancer [56]. It has contributed to cervical cancer progression mediated by Notch signaling [44]. The most interesting observation is its ability to maintain CSC, in breast and head and neck cancer [57, 59]. RhoC knockdown in HNSCC (Head and Neck Squamous Cell Carcinoma) showed a defect in activation of STAT3 in the cells and therefore a reduction in the expression of core stem cell markers like Oct3/4, Sox2, and Nanog [57]. Rosenthal et al. show that RhoC expression impacts the frequency of CSCs in breast cancers. They also show that RhoC expression alone is enough to induce metastasis in even the non-CSC population using mice xenograft [59]. Rho GTPases have also been occasionally implicated in the resistance of tumor cells to therapy.

The other RhoGTPases that have been shown to contribute to tumor progression are Cdc42 and Rac1. Cdc42 inhibition using small molecule inhibitor, AZA197, is reported to suppress the growth of colorectal cancers [63]. There are no mutations reported in the Cdc42 itself, but inhibition of Cdc42 leads to regression of tumors in intestinal cancers which harbour mutations in APC/*β*-Catenin [64], but there is no direct evidence for its involvement in CSC maintenance. However, Rac1 proved important in CSC activity, in both the side population (SP) cells and non-SP cells within the tumor in NSCLA (Non-Small Cell Lung Adenocarcinoma) [65]. In Fanconi Anemia (FA), there are gene mutations that are known to increase tumor invasion in HNSCC via the Rac1 GTPase, acting downstream of the DNA-PK pathway [66]. PREX2, a GEF for RAC1, has been shown to be associated with PTEN pathway, where the suppression of this GEF is necessary for PTEN tumor suppressor activity [67]. This evidence illustrates association of Rho GTPases with CSC and propels the need to study the GTPases in great detail, as they regulate key cellular processes.

The effectors of Rho GTPases, Rho kinases (ROCKs) [36, 68], have also been implicated in tumor progression and metastasis. However, there is no data suggesting their involvement in radiation response. One of the early evidences of nuclear localization of ROCK2 comes from Tanaka et al., where ROCK2 is required for the acetyltransferase activity of p300 [69]. In 2006, Ma et al. show that ROCK2 is important for centrosomal duplication and, hence, important for the maintenance of chromosomal integrity. In tumors, ROCK2 functions as the effector of NPM, a known regulator that controls centrosomal duplication [70]. Hence, deregulation of ROCK2 could have consequences, in the tumor scenario. Another interesting observation published by Wang et al. has broadened the scope of current literature and has added a new dimension in the field, where ROCK2 forms a trimeric complex with BRCA2 and NPM in the centrosomes [71]. In this study, ROCK2 and NPM were identified as binding partners of ROCK2, by mass spectrometric analysis. Deregulated expression of Rho kinases in various tumors and the association of ROCK2 with p300 (epigenetic regulator) and its interaction with the DNA repair pathway indicate the importance of this molecule in cancers and reiterates the need for broadening the perspectives of its study, in the context of CSC and radioresistance. These observations and our unpublished data suggest a possible role for ROCK2 in radiation resistance. Despite convincing reports on their role in various tumor phenotypes, Rho GTPases and their effectors have not been developed further as prognostic markers or therapeutic targets.

## 5. Conclusion

The accumulated data and experimental evidence suggest a role of CSC in therapy resistance, relapse, and metastasis. Their survival and adaptive skills are the factors regulating their maintenance and hence influencing the tumor survival and relapse. In the context of tumor heterogeneity and advent of personalised medicine, it is essential that tumor-specific CSC should be well understood to develop new therapeutic targets.

In addition to enhanced DNA repair activity [12, 14, 19], the CSCs have an expression of efflux proteins which endow protective properties to these cells [72]. The defence mechanisms mostly operative in radiation resistance include DNA damage repair and salvage pathways. It is thus essential to understand the various signaling networks and cross talk that have a protective role in the context of radiation-induced DNA repair. There are several molecular pathways which contribute to the properties of radioresistance in CSC that need to be understood and explored in order to identify new druggable targets.

The GTPases and their effectors may be one such target. Though these molecules have been well studied in the past in the context of metastatic cancers, there is a dearth of literature clearly indicating their role in the context of radioresistance and absolutely none illustrating the role of the effectors in a similar context. However, there is some evidence to implicate their role in DNA repair and radiation in specific cellular contexts, paving the way for focused research on GTPases and their kinases [51, 52, 68] in radioresistance. Atorvastatin is used as GTPases inhibitor to understand their function. It blocks HMG-CoA reductase pathway required to produce geranylgeranyl pyrophosphate (GGPP) and Farnesyl pyrophosphate (FPP). GGPP and FPP are important modulators of Rho GTPases. Interestingly, there are encouraging reports that the incidence of cancer is reduced in statin users [73, 74]. Study indicates that Simvastatin sensitizes the tumor xenotransplants from FaDu (Hypopharynx Squamous cell carcinoma) cells and A431, a vulvar squamous cell carcinoma derived cell line [75]. In breast cancers, it has been found that the statins reduce the rate of tumor recurrence and act as a neoadjuvant in cases which are difficult to treat like the triple negative and inflammatory breast cancers [76]. However, in another study, the statins have been reported to have radioprotective properties in normal tissues through modulation of production of inflammatory cytokines and enhancing DNA repair in the nontarget tissues [77]. These studies and several others suggest the need to understand the role of Rho GTPases and their effectors in therapy resistance. The hope to find newer and better therapeutic targets, for cancer treatment, nudges us to explore newer signaling pathways. Rho GTPases and their effectors have a significant contribution to cancer progression, and it is only apt that these molecules should be further explored in the context of CSC, to aid in better cancer treatment.
